# Chemokines and Inflammatory Mediators Interact to Regulate Adult Murine Neural Precursor Cell Proliferation, Survival and Differentiation

**DOI:** 10.1371/journal.pone.0025406

**Published:** 2011-09-23

**Authors:** Alisa Turbic, Soo Yuen Leong, Ann M. Turnley

**Affiliations:** Centre for Neuroscience, The University of Melbourne, Parkville, Victoria, Australia; Emory University, United States of America

## Abstract

Adult neural precursor cells (NPCs) respond to injury or disease of the CNS by migrating to the site of damage or differentiating locally to replace lost cells. Factors that mediate this injury induced NPC response include chemokines and pro-inflammatory cytokines, such as tumor necrosis factor-α (TNFα) and interferon-γ (IFNγ), which we have shown previously promotes neuronal differentiation. RT-PCR was used to compare expression of chemokines and their receptors in normal adult mouse brain and in cultured NPCs in response to IFNγ and TNFα. Basal expression of many chemokines and their receptors was found in adult brain, predominantly in neurogenic regions, with OB≫SVZ>hippocampus and little or no expression in non-neurogenic regions, such as cortex. Treatment of SVZ-derived NPCs with IFNγ and TNFα (alone and in combination) resulted in significant upregulation of expression of specific chemokines, with CXCL1, CXCL9 and CCL2 most highly upregulated and CCL19 downregulated. Unlike IFNγ, chemokine treatment of NPCs *in vitro* had little or no effect on survival, proliferation or migration. Neuronal differentiation was promoted by CXCL9, CCL2 and CCL21, while astrocyte and total oligodendrocyte differentiation was not affected. However, IFNγ, CXCL1, CXCL9 and CCL2 promoted oligodendrocyte maturation. Therefore, not only do NPCs express chemokine receptors, they also produce several chemokines, particularly in response to inflammatory mediators. This suggests that autocrine or paracrine production of specific chemokines by NPCs in response to inflammatory mediators may regulate differentiation into mature neural cell types and may alter NPC responsiveness to CNS injury or disease.

## Introduction

Effective neural repair is likely to require a multi-factorial approach, including blockage of neuronal death and replacement of lost cells by differentiation of neural precursor cells (NPCs). Following CNS damage NPCs can migrate from their normal location in the subventricular zone (SVZ) to the site of damage [1], [2], at least partly due to the inflammatory response and production of cytokines and chemokines. Chemokines have been widely studied with respect to immune cell migration but various members of the chemokine family are also expressed in the normal CNS, making them prime candidates for regulating both injury-induced as well as normal NPC function.

Chemokines are primarily known for regulating chemoattraction of immune cells to sites of tissue damage. The family is divided into 4 groups based on the relative position of the first N-terminal cysteine (CC, CXC, CX3X and XC or δ families) [3]. They signal through G-protein coupled receptors (CCR, CXCR, CX3CR, CR). Their expression can be induced or upregulated under inflammatory conditions. However, many chemokines and their receptors are also constitutively expressed, both during development and in some areas of the adult nervous system. They have been reported to have widespread non-immunological effects in the CNS, including regulation of neural cell proliferation, migration, survival and synaptic transmission and can act in a paracrine or autocrine manner [3], [4]. Some, such as CXCL12, also have a developmental role and are reported to be involved in neuronal precursor migration in a number of areas, such as cerebellum [4]. In response to injury, astrocytes and microglial cells appear to be a major source of chemokines that attract leukocytes to the injury site [5], [6], [7]. There is little information available about region specific expression of these factors in adult CNS under normal conditions. Neurospheres derived from embryonic or adult mouse NPCs express several chemokine receptors, with some differences in E17 versus adult expression. E17 neurospheres expressed CCR1–3, 5, 7–10 and CXCR1–4 & 6, while the adult spheres expressed CCR1–8, 10 and CXCR1–6 [8] and human embryonic NPCs expressed CCR3 and CXCR4 [9]. CXCL12 via CXCR4 promotes NPC migration, proliferation or survival [8], [10], [11]), although it has also been reported to inhibit proliferation and promote quiescence [9].

Inflammatory mediators, such as IFNγ and TNFα are regulators of chemokine and chemokine receptor expression in many tissues [12], [13]. This includes the CNS, in which IFNγ upregulates CCL2 [14], CXCR4 and CCR5 [15] in astrocytes. IFNγ plus TNFα in conjunction with CCL2/CCR2 promote migration of NPCs to the site of cytokine injection [7] and IFNγ is a potent inhibitor of NPC proliferation that promotes neuronal differentiation [16]. Whether this is a direct effect or may be mediated by chemokines is not yet known.

We have now examined chemokine and chemokine receptor expression in neurogenic versus non-neurogenic regions of the adult CNS, as well as regulation by IFNγ and TNFα in NPCs. The role of selected chemokines alone or in combination with IFNγ, on neural precursor proliferation, survival, migration and differentiation was also examined.

## Materials and Methods

### Reagents

Chemokines: Recombinant mouse MIP-3β/CCL19, 6Ckine/CCL21, TECK/CCL25 and LIX/CXCL5 were purchased from R&D Systems. Recombinant mouse JE/CCL2, MIP-3α/CCL20, KC/CXCL1, MIG/CXCL9, CXCL16 and human IL-8/CXCL8 were purchased from Peprotech Inc (Princeton, NJ, USA). Recombinant mouse Tumor Necrosis Factor-α (TNFα) was from Millipore (Melbourne, Australia) and recombinant mouse Interferon-γ (IFNγ) from BD Pharmingen (Sydney, Australia).

### Ethics statement

Animal use was approved by the University of Melbourne Animal Ethics Committee and experiments were performed in accordance with National Health and Medical Research Council of Australia guidelines (approval #06218).

### Neurosphere culture

Neurospheres were generated from cells isolated from the subventricular zone of 6–8 week old C57BL/6 mice and cultured in proliferation media consisting of DMEM/F12 (GibcoBRL/Invitrogen) containing 0.6% glucose, 3 mM NaHCO3, 5 mM HEPES, 2 mM L-glutamine, 0.1 mg/ml apo-transferrin, 25 µg/ml insulin, 60 µM putrescene, 30 mM sodium selenite, 20 nM progesterone and 1% BSA (all from Sigma), supplemented with EGF (20 ng/ml; Peprotech) and FGF2 (10 ng/ml; Peprotech), as previously described [17]. Cells were cultured for 6–7 days and used up to passage 10.

### RT-PCR for chemokine and chemokine receptor expression analyses

Olfactory bulb, subventricular zone, hippocampus and cortex tissue was collected from 8 week old C57Bl/6 adult mice. Neurosphere cultures were treated with IFNγ (100 U/mL), TNFα (100 U/mL) or combination of both for 24 h and cells collected. Total RNA was isolated from tissue and cultured neurospheres using an RNeasy Mini Kit (Qiagen, Australia) according to the manufacturer's instructions. The cDNA was prepared from 2.0 µg RNA using 50 µg oligo-(dT)_20_ (GeneWorks, Australia) and SuperScript Reverse Transcriptase (Invitrogen, Australia) following the manufacturer's protocol. For semi-quantitative RT-PCR, cDNA was subsequently amplified using Hot-StarTaq DNA Polymerase (Qiagen, Australia) and primers as listed in Table 1 using a PCR Express machine (Thermo Scientific, USA). The PCR program consisted of initial denaturation at 95°C for 15 min, followed by 40 cycles of denaturation at 94°C for 30 s, annealing at 58°C for 30 s and extension at 72°C for 30 s, with a final extension at 72°C for 10 min for all primers. A 10 µl aliquot of each PCR product was size-separated by electrophoresis in 2% agarose gels containing Sybr Safe (Invitrogen, Australia) and imaged by digital gel documentation using an LAS-3000 machine (Fuji, Japan). Densitometric analysis of level of expression was performed using ImageJ software (NIH), normalised against GAPDH levels.

**Table 1:** Primer sequences used for RT-PCR.

Gene	Forward primer (Sequence 5′→3′)	Reverse primer (Sequence 5′→3′)
**CCL2**	CCC AAT GAG TAG GCT GGA GA	CCT TAG GGC AGA TGC AGT TT
**CCL19**	TTC ACC ACA CTA AGG GGC TA	GCC ACA GAG AGA TGG TGT TC
**CCL20**	CTT GCT TTG GCA TGG GTA CT	TCA GCG CAC ACA GAT TTT CT
**CCL21**	GGG GAA CCT CTA AGT CTG GA	TGC TGT CTC CTT CCT CAT TC
**CCL25**	GGC GAT GAG AAT CTT GAC AG	TAG CCA ATA GCT CCT TGG TG
**CCR1**	ATG GAG ATT TCA GAT TTC	TCA GAA GCC AGC AGA GAG
**CCR2**	ATG TTA CCT CAG TTC ATC CAC GGC	GTA ATG GTG ATC ATC TTG TTT GGA
**CCR3**	ATG GCA TTC AAC ACA GAT G	CAA CTA AAA CAC CAC AGA
**CCR4**	ATG AAT GCC ACA GAG GTC	TTA CAA AGC GTC ACG GAA G
**CCR5**	ATG GAT TTT CAA GGG TCA G	TCA TAA ACC AGT AGA AAC
**CCR6**	TAG GAC TGG AGC CTG GAT AAC CAC	TAG GGC TTG AGA TGA TGA TGG AGA
**CCR7**	ATG GAC CCA GGG AAA CCC A	CTA CGG GGA GAA GGT TGT G
**CCR8**	ATG GAT TAC ACG ATG GAG	CAC TCC CCT TAC AAG ATG TC
**CCR9**	GTC ACC TTG GGG TTT TTC CT	ACA TGG CAT AAG CGT CAA CA
**CCR10**	ATG GGG ACC AAG CCC ACA GAG	CTA GTT GTC CCA AGA GAG AC
**CCR11**	GTT TCT CTG ACC CCA CTG	GCA GGA AAC ACA GGT TTG
**CXCL1**	ATG ATC CCA GCC ACC CGC TC	TTA CTT GGG GAC ACC TTT TAG C
**CXCL2**	GAT ACT GAA GAG CGG CAA GTC	AAG ACA CAT CCA GAC ACC GTT
**CXCL3**	AGA TCT CAC CAC AGC CCT TC	AAC CCT TGG TAG GGT GTT CA
**CXCL4**	CCC TAG ACC CAT TTC CTC AA	AGA AAC AAC AGG CCC AGA AG
**CXCL5**	GCC CTA CGG TGG AAG TCA TA	AGT GCA TTC CGC TTA GCT TT
**CXCL7**	CAC TGC TCT TCA TTA TGG GC	GGT CAG TAA CCT TCC AAG
**CXCL8**	TCT TCT TGC TCT GTT GTC TGC CCT	GCG GCG TTC GCA AGT ATC TTC AAT
**CXCL9**	ATG AAG TCC GCT GTT CTT TTC C	GTC TCT TAT GTA GTC TTC CTT G
**CXCL10**	AGT AAC CCA AGT GCT GCC GTC	CTC CAG TTA AGG AGC CCT TTT AG
**CXCL11**	ATG AAC AGG AAG GTC ACA GC	GAT GTC ACA TGT TTT GAC GC
**CXCL12**	GTC CTC TTG CTG TCC AGC TC	TTT CAG ATG CTT GAC GTT GG
**CXCL13**	ATG AGG CTC AGC ACA GCA AC	TCA GGC AGC TCT TCT CTT AC
**CXCL14**	AGC ATG AGG CTC CTG G	CTA TTC TTC GTA GAC C
**CXCL15**	ATG GCT GCT CAA GGC TGG TC	TTA GGC ATC ACT TGT C
**CXCL16**	ATG AGG CGG GGC TTT GGA C	CTA GGG TCT TGG TTC AAC AG
**CXCR1**	GCT GCC CAC TGG AGA TTA TTT C	TAT GCC TGG CGG AAG ATA GC
**CXCR2**	ATG GGA GAA TTC AAG GTC	TTA GAG GGT AGT AGA GGT G
**CXCR3**	GCA AAT GTG GAT GTT GTT CA	ATG GTG TTG TCC TTG TTG CT
**CXCR4**	ATG GAA CCG ATC AGT GTG	GTT AGC TGG AGT GAA AAC
**CXCR5**	TGG ATG ACC TGT ACA AGG AAC TGG	AAC GGG AGG TGA ACC CTC TAG AGG
**CXCR6**	ATG CCA TGG ATG ATG GGC	CTA CAA TTG GAA CAT ACT GG
**CXCR7**	CAG GCT AAG ACC ACA GGC TA	CCA CAC CAA GAT GCA TAC AA
**CX3CL1**	ACT CCA GCC ATG GCT CCC TC	TCA CAC TGG CAC CAG GAC G
**CX3CR1**	CAC CAT GTC CAC CTC CTT CCC TG	CTT CAG AGC AGG AGA GAC
**XCL1**	CAA GAC CTC AGC CAT GAG AC	GGA ACA GTT TCA GCC ATG TT
**XCR1**	ATG GAG TCC TCT ACA G	CTC CTC TCA GTA GAA G
**GAPDH**	TCC ACC ACC CTG TTG CTG TA	ACC ACA GTC CAT GCC ATC AC

For quantitative RT-PCR (qPCR), cDNA was prepared as above, except that RNA was DNase treated using Ambion DNA-*free* DNase Treatment and Removal Reagent (Applied Biosystems, Australia). For each sample, real-time qPCR was conducted in triplicate using Platinum SYBR Green qPCR SuperMix-UDG (Invitrogen, Australia) and primers as listed in Table 2 using the Rotor-Gene 6000 PCR system (Corbett Research, Australia). “No template controls”, in which no cDNA template is added, were also included for each master mix prepared. Cycling conditions were as follows: 50°C for 2 min (UDG incubation), 95°C for 2 min, followed by 40 cycles of 95°C for 15 s, 60°C for 30 s and 72°C for 20 s. Dissociation (melting) curve was added as the final step (72°C–95°C) to ensure that only the specific target was amplified in the PCR reaction. Results were analyzed with Roto-Gene 6 Software (Corbett Research, Australia) and the relative quantitation was achieved by applying the comparative C_T_ method (ΔΔC_T_) [18] whereby the mRNA levels were normalized against the level of housekeeping gene glyceraldehyde-3-phosphate dehydrogenase (GAPDH) mRNA. All samples from individual experiments were prepared and run at the same time in the same machine to allow comparison of relative levels of different transcripts within each experiment. Transcripts were considered to be not expressed if they had C_T_ values >32. For analysis of CXCR expression in CNS tissues, all samples were compared to levels of CXCR5 in the olfactory bulb, which was expressed at the lowest level detectable and given a reference value of 1. For analysis of chemokine expression in neurospheres induced by IFNγ and TNFα, for each chemokine the reference value of 1 was given to the expression level under basal conditions (for CXCL1 and CCL2) or if not expressed at basal levels (C_T_>32), the condition under which lowest expression was detected (TNFα for CXCL9 and IFNγ for CXCL13).

**Table 2:** Primer sequences used for qPCR.

Gene	Forward primer (Sequence 5′→3′)	Reverse primer (Sequence 5′→3′)
**CCL2**	GGC CTG CTG TTC ACA GTT GC	CCT GCT GCT GGT GAT CCT CTT
**CXCL1**	GAC CAT GGC TGG GAT TCA CC	CCA AGG GAG CTT CAG GGT CA
**CXCL9**	CCG AGG CAC GAT CCA CTA CA	CGA GTC CGG ATC TAG GCA GGT
**CXCL13**	TTC TGG AAG CCC ATT ACA CAA A	CCA TTT GGC ACG AGG ATT CA
**CXCR1**	ACC CGA TCC GTC ATG GAT GT	GGC CAG GTA TCG GTC CAC AC
**CXCR2**	GCT GTC CCA TGC CAC TCA GA	GGC AAG GTC AGG GCA AAG AA
**CXCR3**	AAC CTT CCT GCC AGC CCT CT	CGA AAA CCC ACT GGA CAG CA
**CXCR4**	TCC GGG ATG AAA ACG TCC AT	CAG CCG GTA CTT GTC CGT CA
**CXCR5**	CTG GAC ATG GGC TCC ATC A	GCT GTA GGC CAC AGG CAT GA
**CXCR6**	CCC TGG CTG ACC TGG TGT TT	TGC AGG TGA GAG TGA GCA TGG
**CXCR7**	AGC AGC GAC TGC ATT GTG GT	TGC AAT GGC CAG GTT CAA GA
**GAPDH**	TCC CAG AGC TGA ACG GGA AG	TCA GTG GGC CCT CAG ATG C

### MTS proliferation assay

Neurospheres were pre-treated with IFNγ (100 U/mL) for 24 h prior to setting up the assay then dissociated into single cells and plated at 5×10^4^ cells/well in proliferation media. Chemokines (100 ng/mL) and IFNγ were added at time of plating and at 2-day intervals thereafter. At day 7, cells were collected and transferred to 96-well plates containing 100 µl of media from 24-well plates and viable cell number assessed using the CellTiter 96 Aqueous One Solution Reagent (Promega, Madison, WI, USA), according to the manufacturer's instructions. The background absorbance of the media only control was subtracted from all samples and then the absorbance values of the treated samples were all compared to the untreated (basal) samples (expressed as a percentage of untreated) using the following formula: Treated(Absorbance-background)/Basal(Absorbance-background)×100 and the results from three independent experiments using three different neurosphere lines were used to calculate the mean+/−SEM.

### Migration, proliferation and cell death assays

Neurospheres were pre-treated with IFNγ 100 U/mL for 24 h prior to setting up the assay. Whole spheres (80–100 µm size) were plated onto human plasma fibronectin (0.05 mg/mL, Chemicon) coated 96-well plates in proliferation media with or without chemokines (100 ng/mL) or IFNγ (100 U/mL). Cells were allowed to adhere and migrate for 24 h prior to assessing effects on proliferation, cell death and migration. To assess the percentage of proliferative cells, cells were fixed and immunostained using rabbit anti-Ki67 antibody (1∶200, Lab Vision) with goat anti-rabbit Cy2 (1∶1000; Jackson Immunoresearch) and DAPI (1∶3000). To assess the extent of cell death, a TUNEL assay was performed using an In Situ Cell Death Detection kit (Roche, Sydney Australia) according to the manufacturer's instructions. For each experiment, the number of Ki67 or TUNEL positive cells and DAPI stained cells was counted for at least 10 fields/well, with 3 wells/plate per condition and the percentage of cells that were Ki67 or TUNEL positive was calculated.

The extent of migration was quantified by tracing around individual neurospheres using ImageJ software (Wayne Rasband, Research Services Branch, National Institute of Mental Health, Bethesda, Maryland, USA) and the macro “Hull and Circle” (NIH, USA) written by Audre Karperien, Charles Sturt University, Australia and Thomas R. Roy, University of Alberta, Canada. To account for variations in cell number per neurosphere, the area of migration of each neurosphere was divided by the number of DAPI positive cells per neurosphere.

For each of the assays above, to enable comparison of results between different experiments and neurosphere lines, the result of the basal condition in each assay was set to 100% and the results of the treated conditions were expressed as a percentage of basal using the following formula: Result(Treated)/Result(Basal)×100. Three independent experiments using three different neurosphere lines were then used to calculate the mean+/−SEM.

### Neurosphere Differentiation assay

Neurospheres were dissociated into single cells and plated at 1.5×10^5^ cells per well onto poly-L-ornithine (Sigma) and laminin (Invitrogen) coated 8-well chamber glass slides (BD Falcon). Differentiation media was the same as proliferation media but with 2% FBS added and no EGF or FGF2. Chemokines (10 or 100 ng/mL) and IFNγ (100 U/mL), alone and in combination, were added to the media at time of plating and cells were fixed at 3 and 7 days. For the 7 day time point fresh media was added at day 3. Cells were immunostained for neuronal (mouse anti-βIII-tubulin;1∶3000, Promega), astrocyte (rabbit anti-GFAP; 1∶500, Dako) and oligodendrocyte (mouse anti-O4; 1∶200, Chemicon) markers essentially as previously described [16]. Secondary antibodies used were goat anti-mouse-Cy3 and goat anti-rabbit-Cy2 (1∶1000, Jackson Immunoresearch), goat anti-mouse IgM Alexa Fluor 488 (1∶300, Molecular Probes) and 4′, 6-diamidino-2-phenylindole (DAPI, 1∶3000; Sigma) was used to label nuclei. After washing with PBS coverslips were mounted onto slides using fluorescent mounting medium (Dako, Carpentaria, USA).

Immunolabelled cells were counted from 10 fields/well, for 3 wells for each condition and the results expressed as a percentage of total, DAPI-labeled cells in each experiment. For O4 positive cells, in addition to total O4 cell numbers, counts were performed based on morphology: cells were classified as precursor cells (bipolar or tripolar cells), pro-oligodendrocytes (a large number of thin processes) or oligodendrocytes (flattened membranous growth).

To enable comparison of results between different experiments and neurosphere lines, the result of the basal condition in each assay was set to 100% and the results of the treated conditions were expressed as a percentage of basal using the following formula: Result(Treated)/Result(Basal)×100. At least three independent experiments using three different neurosphere lines were then used to calculate the mean+/−SEM.

### Statistical analysis

All results are presented as mean+/−SEM, combined from at least *n* = 3 individual experiments. Group data was analyzed by ANOVA, followed by Tukey post-hoc comparisons or Dunnet's post-hoc comparison to control, using GraphPad Prism 4.0 software. Significance was set as *p*<0.05.

## Results

### Chemokine and receptor expression in neurogenic and non-neurogenic regions of the adult CNS

**Figure 1:**
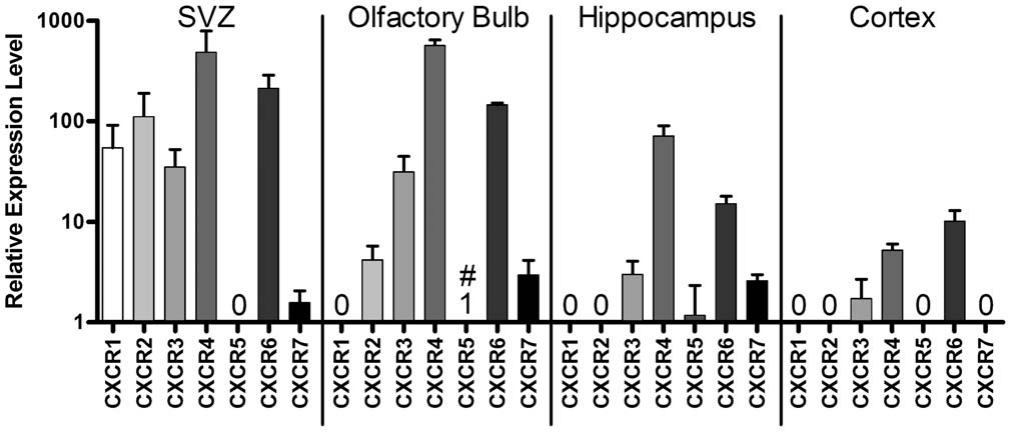
Expression of CXCR chemokine receptors in neurogenic versus non-neurogenic regions of adult brain. qPCR analysis of chemokine receptor CXCR1–7 expression in neurogenic subventricular (SVZ), olfactory bulb, and hippocampus and non-neurogenic cortex. Due to large differences in the level of expression of the different receptors, results are presented using a log_10_ scale and show mean+/−SEM of 3 independent experiments in which all samples were run together and normalized against GAPDH. All samples were compared to expression of CXCR5 in the olfactory bulb as the reference sample (denoted by #), which was expressed at just detectable levels (C_T_<32) and given a value of 1. Receptors that were not expressed (C_T_≥32) are denoted by 0 in their respective columns.

Neurogenic regions of the adult brain (SVZ and hippocampus) have previously been shown to express the chemokine receptors CXCR3, CXCR4, CCR1, CCR2 and CCR5 [19]. We have extended these studies to analyze expression of CXC chemokines, CXC receptors, CCR receptors and CX3C and XC chemokines and receptors in neurogenic regions and the olfactory bulb, the destination of SVZ-derived NPCs versus non-neurogenic cortex by qPCR (Fig. 1) and semi-quantitative RT-PCR (Table 3). The olfactory bulb expressed the majority of receptors and ligands tested, most at high to very high levels. The neurogenic SVZ and hippocampus expressed different subsets of receptors and chemokines, some of which overlapped (e.g. CXCR6) and some of which were different (e.g. CXCR2 in SVZ but not hippocampus). Expression of the CXC and CC receptors was largely restricted to neurogenic regions, with only CXCR3, CXCR4, CXCR6, CCR2, CCR6 and CCR9 expressed at low levels in cortex. CX3C receptor was widely expressed as were the majority of CXC ligands, while high CXC3L1 expression was restricted to neurogenic regions. Overall, the olfactory bulb expressed the most chemokines and receptors at the highest levels and the cortex expressed the fewest and at the lowest levels.

**Table 3:** Summary of chemokine ligand and receptor expression in adult brain.

	SVZ	OB	Hipp	Cortex
**CXCR1**	++	−	−	−
**CXCR2**	+++	+	−	−
**CXCR3**	++	++	+	+/−
**CXCR4**	+++	++++	++	+
**CXCR5**	−	+/−	−	−
**CXCR6**	+++	+++	++	++
**CXCR7**	+/−	+	+	−
**CCR1**	+	+	+	−
**CCR2**	−	++++	+	+
**CCR3**	−	++	+	−
**CCR4**	++	+++	−	−
**CCR5**	+	++	+/−	−
**CCR6**	+/−	++++	+++	++
**CCR7**	+++	++++	−	−
**CCR8**	+	++	+	−
**CCR9**	+	++++	+	+
**CCR10**	−	+++	+	−
**CCR11**	−	+/−	−	−
**CXCL1**	+	+	++++	++
**CXCL2**	+/−	++++	+/−	−
**CXCL3**	−	−	−	−
**CXCL4**	+/−	++	+	+
**CXCL5**	+++	++++	−	+
**CXCL7**	++++	++++	++++	++
**CXCL8**	−	+/−	−	−
**CXCL9**	−	++	+	−
**CXCL10**	+	++++	+++	++
**CXCL11**	++	+++	+++	+
**CXCL12**	+	+/−	++	++
**CXCL13**	+++	−	−	+
**CXCL14**	+++	+++	+++	++
**CXCL15**	+	++	++	++
**CXCL16**	+++	+++	+++	++
**CX3CL1**	+++	+++	+++	+
**CX3CR1**	++	+++	++++	+++
**CCL2**	−	+	+/−	−
**CCL19**	+/−	+	++	+
**CCL20**	−	−	−	−
**CCL21**	+/−	+/−	++	+
**CCL25**	−	−	++	+
**XCL1**	−	++	−	−
**XCR1**	+	++	−	−

SVZ, Subventricular zone; OB, olfactory bulb; Hipp, hippocampus; Ctx, cortex.

Expression levels: − none; +/− very low; + low; ++ moderate, +++ high; ++++ very high.

### Chemokine and chemokine receptor expression in neurospheres in response to inflammatory cytokine stimulation

**Figure 2:**
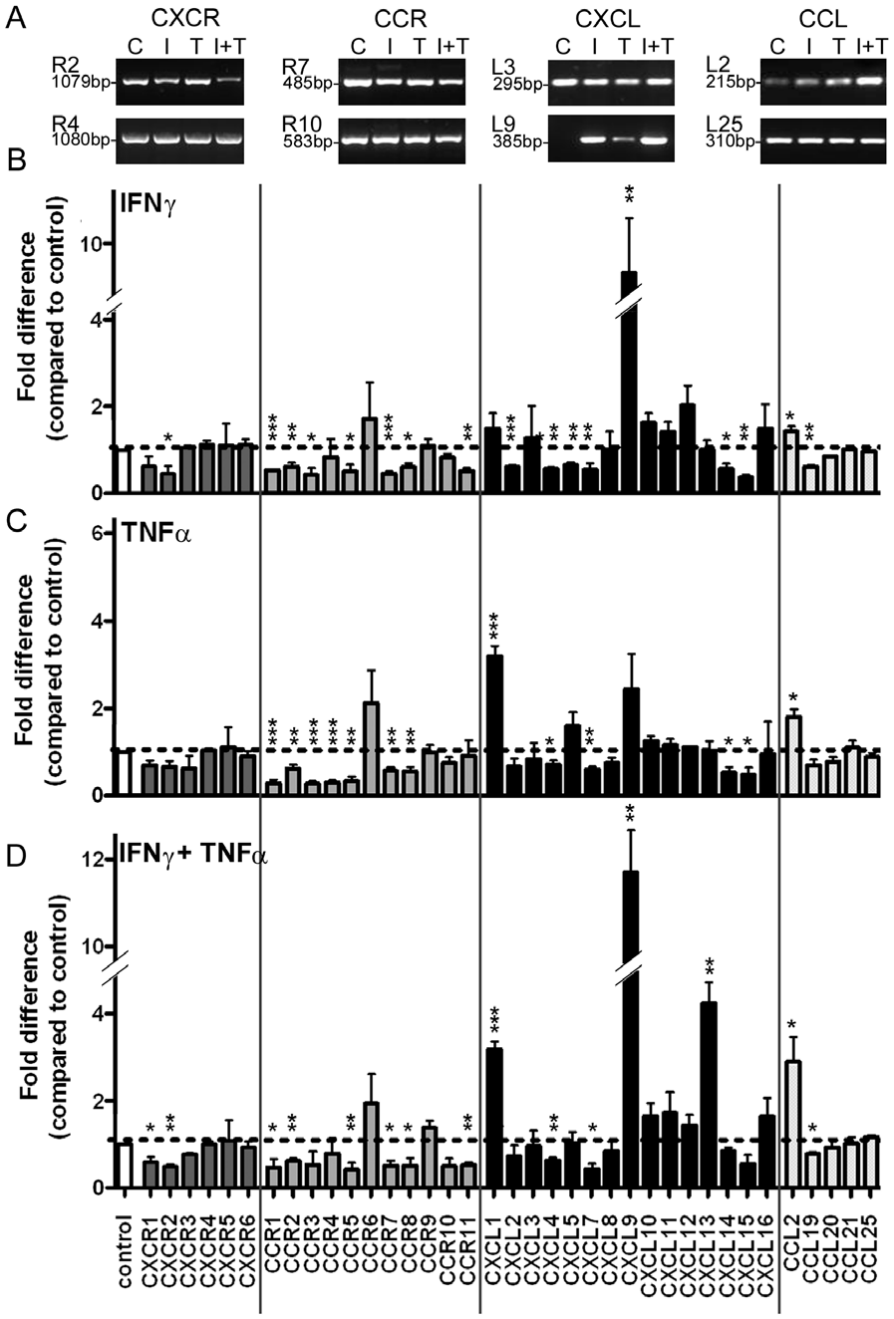
Regulation of chemokine and chemokine receptor expression in adult NPCs by IFNγ and TNFα. (A) Selected representative gels of RT-PCR for chemokine (CXCL and CCL) and chemokine receptor (CXCR and CCR) expression in adult neurospheres under control (C) conditions or treated with 100 U/ml IFNγ (I), 100 U/ml TNFα (T) or IFNγ plus TNFα (I+T). (B–D) The relative expression level of each chemokine or receptor in response to addition of (B) IFNγ, (C) TNFα or (D) IFNγ plus TNFα was normalized by densitometric analysis to control expression levels for each chemokine or receptor and expressed as fold difference compared to control. Results show mean+/−SEM of at least n = 3 individual experiments; *p<0.05, **p<0.01, ***p<0.001.

Previous studies have examined the expression of chemokines and/or chemokine receptors in embryonic and adult neural precursor cells/neurospheres [8], [9], [20], [21]. Adult neurospheres expressed almost all CXC and CCR receptors, as well as CX3CR1 [8], under basal culture conditions, a finding which we have confirmed (Fig. 2). We also show that adult neurospheres express all CXC ligands (CXCL1–CXCL16) and 5 CCL ligands we examined (CCL2, CCL19, CCL20, CCL21, CCL25).

**Figure 3:**
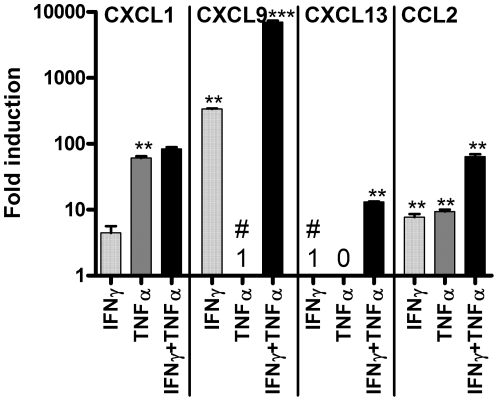
Confirmation of chemokine induction by qPCR. qPCR was used to confirm the upregulation or induction of chemokine expression by IFNγ and/or TNFα. Due to large differences in the level of expression of the different chemokines, results are presented using a log_10_ scale and show mean+/−SEM of 3 independent experiments in which all samples were run together and normalized against GAPDH. CXCL1 and CCL2 were detectable in the neurospheres under basal conditions, therefore their expression following incubation with IFNγ and TNFα was compared to the level of expression under basal conditions (which was set to 1). CXCL9 and CXCL13 were not expressed at detectable levels under basal conditions (C_T_≥32). CXCL9 was detectable following addition of TNFα, which was used as the reference condition in this case (and set to 1; denoted by #) for comparison with expression induced by IFNγ and IFNγ+TNFα. CXCL13 was not detectable with TNFα treatment (denoted by 0) but was induced by IFNγ which was used as the reference condition (and set to 1; denoted by #) for comparison with levels of expression induced by TNFα plus IFNγ.

As inflammatory cytokines, such as IFNγ and TNFα have been shown to regulate chemokine and chemokine receptor expression in a variety of neural and non-neural cell types [7], [12], [13], [14], [15] and the response of neural precursor cells to neural damage includes effects of inflammatory cytokines [7], we examined whether IFNγ and TNFα alone and in combination modulated expression of chemokines and their receptors in neurospheres (Fig. 2). The inflammatory cytokines regulated overall chemokine and receptor expression levels in the neurospheres: IFNγ *F*_(41, 84)_ = 15.22, *p*<0.0001; TNFα *F*_(41, 84)_ = 4.79, *p*<0.0001; IFNγ plus TNFα *F*_(41, 84)_ = 13.99, *p*<0.0001. In general however, they had little or no effect on the majority of the CXC and CC receptors and several of the CXC ligands. Conversely, IFNγ alone markedly increased expression of CXCL9, which was partially augmented by co-addition of TNFα. TNFα alone significantly increased expression of CXCL1, while the combination of IFNγ and TNFα was required to increase expression of CXCL13. CCL2 expression was modestly increased by IFNγ or TNFα alone and enhanced by the combination. The induction/upregulation of expression of CXCL1, CXCL9, CXCL13 and CCL2 by the inflammatory cytokines was confirmed by repeating the experiments with new neurosphere lines and performing qPCR (Fig. 3).

Several chemokines have been reported to have widespread non-immunological effects in the CNS, including regulation of neural cell proliferation, migration, survival and differentiation and can act in a paracrine or autocrine manner [3], [4], [11], [22], [23]. Although most chemokines and their receptors are expressed by neural precursor cells, most research on their function in CNS cells, including NPCs, has to date focused primarily on the macrophage attracting chemokine CXCL12 (previously known as SDF1; stromal-derived factor-1) and its receptor CXCR4 or CCL2 (MCP-1, monocyte chemoattractant protein), particularly with regard to migration. We have therefore examined the effect of several other chemokines on neural precursor cells: CXCL1 and CXCL9, whose expression was upregulated in neural precursor cells by IFNγ or TNFα, as well as CXCL5 and CXCL8, which, like CXCL1, signal through CXCR2. We also examined CXCL16, expression of which wasn't significantly modulated by inflammatory cytokines and CCL2, which has previously been shown to have multiple effects on neural precursor cells. We also examined the effect of CCL19, 20, 21 and 25, which have been reported to play a role in neuroinflammation [24].

### Effect of IFNγ and chemokines on neurosphere growth

**Figure 4:**
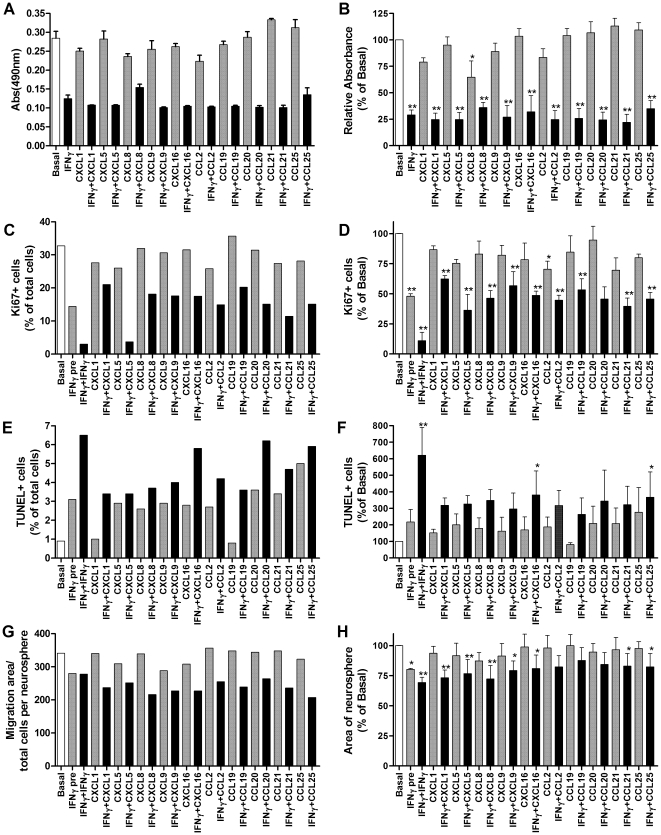
Regulation of NPC proliferation survival and migration by chemokines and IFNγ. (A,B) Effect on neurosphere growth. Neurospheres were grown for 7 days in the presence of chemokines (100 ng/ml) and/or IFNγ (100 U/ml) and neurosphere growth determined by assessment of viable cell number using the MTS assay. Results of a representative experiment are shown in A and the combined results of three independent experiments, which are expressed as a percentage of neurosphere growth under basal conditions are shown in B. Effect on NPC proliferation (C,D), survival (E,F) and migration (G,H): neurospheres were pre-treated for 24 hrs with IFNγ then plated onto fibronectin in proliferation medium alone (IFNγ pre), with IFNγ alone (IFNγ+IFNγ), with chemokines alone or with chemokines plus IFNγ for 24 hrs. Basal controls were not pre-treated with IFNγ nor treated with any factor at the time of plating. Representative results from individual experiments are shown in C, E and G and the combined results of three independent experiments, expressed as a percentage of basal, are shown in D, F and H. (C,D) Effect on proliferation was assessed by counting the percentage of cells immunostained for the proliferation marker Ki67. (E,F) Effect on cell survival was assessed by counting the percentage of cells that were labeled by TUNEL staining. (G,H) Effect on cell migration was assessed by measuring the area of spread of cells from individual neurospheres and dividing by cell number per neurosphere. Results are expressed as mean+/−sem, *p<0.05, **p<0.01.

We have previously shown that IFNγ is not toxic but inhibits proliferation of neurospheres [16], [25]. To determine whether the chemokines selected above also modulate neurosphere growth, alone or in combination with IFNγ, neurospheres were grown for 7 days in proliferation media with chemokines+/−IFNγ and neurosphere growth determined by assessment of viable cell number using the MTS assay (Fig. 4a,b). Significant inhibition of neurosphere growth was observed in the presence of IFNγ, under all conditions tested (ANOVA *F*_(19, 38)_ = 22.49, *p*<0.0001). The chemokines in general had little or no effect on neurosphere growth by themselves, although CXCL8 produced a modest decrease (ANOVA *F*_(10, 22)_ = 3.2, *p* = 0.01).

### Effect of IFNγ and chemokines on neural precursor cell proliferation, survival and migration

Neurospheres were pretreated for 24 hrs with IFNγ to induce chemokines and IFNγ signaling pathways and then plated onto a fibronectin substrate in proliferation medium containing IFNγ and/or chemokines for 24 hrs. Cell proliferation, survival and migration were then assessed by quantitation of the percentage of Ki67 positive cells, TUNEL positive cells and the area covered by the neural precursor cells from each neurosphere, respectively. Similar to growth of neurospheres, pretreatment of plated neurospheres with IFNγ decreased the percentage of proliferative cells (from approximately 30±3% under basal conditions to 15±1% Ki67+ cells with IFNγ pretreatment; ANOVA *F*_(20, 40)_ = 15.54, *p*<0.0001) and this was further reduced by continued treatment with IFNγ alone to 4±2% (Fig. 4c,d). Chemokines by themselves had no significant effect on the percentage of proliferative cells (ANOVA *F*_(10, 22)_ = 1.09, *p* = 0.413, ) except for CXCL5 which partially abrogated the decrease in Ki67 positive cells induced by IFNγ. While proliferation was still significantly decreased by IFNγ in the presence of chemokines, compared to basal conditions, the chemokines significantly increased the percentage compared to IFNγ treatment alone (ANOVA *F*_(11, 24)_ = 7.214, *p*<0.0001). This may have largely been due to an effect on cell survival. Pretreatment of neurospheres with IFNγ alone had no effect on cell death but continued administration of IFNγ alone for a further 24 hrs induced significant neural precursor cell death (Fig. 4e,f). However, continued administration of IFNγ in the presence of CXCL1, CXCL5, CXCL8, CXCL9, CCL2, CCL19 and CCL21 did not produce significant cell death (ANOVA *F*_(20, 42)_ = 0.865, *p* = 0.63) and in the presence of CXCL16 and CCL25 only modest cell death, which was less than induced by IFNγ alone.

When neurospheres are plated onto the fibronectin substrate, they adhere and the cells then migrate away in a non-directed fashion. To determine whether the chemokines affected such basal migration, the area of each neurosphere was measured 24 hrs after plating. None of the chemokines tested had any effect when compared to non-treated neurospheres (ANOVA *F*_(10, 22)_ = 0.234, *p* = 0.99), although IFNγ pretreatment produced a modest decrease in migration, which was not abrogated by the chemokines (Fig. 4g,h).

### Effect of IFNγ and chemokines on neural precursor cell differentiation

**Figure 5:**
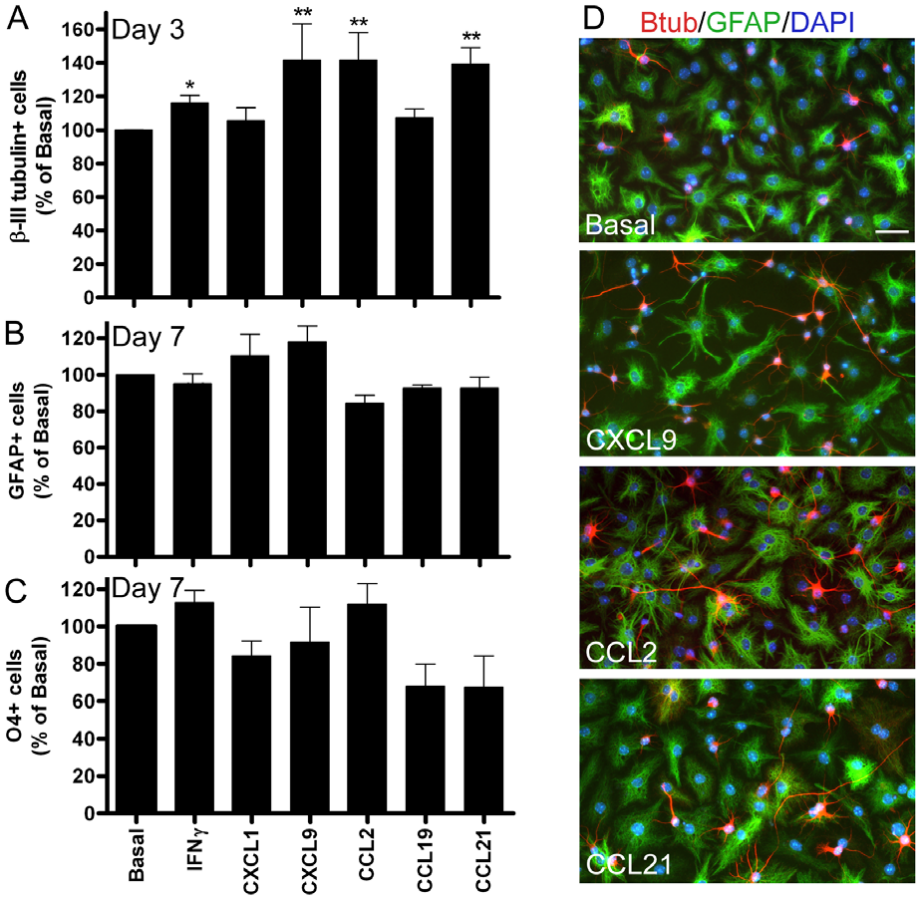
Effect of selected chemokines on NPC differentiation. (A) The effect of chemokines (10 ng/ml) on neuronal differentiation as assessed by quantifying the relative percentage of cells that expressed the neuronal marker βIII-tubulin at 3 days under treatment conditions compared to the basal control. (B) The effect on astrocyte differentiation was assessed by counting the percentage of cells immunostained for the astrocyte marker GFAP at 7 days, expressed as a relative percentage compared to the basal control. (C) The effect on oligodendrocyte differentiation was assessed by counting the number of cells immunostained for the oligodendrocyte marker O4 at 7 days and expressing this as a percentage relative to the basal control. (D) Representative micrographs of βIII-tubulin, GFAP and DAPI labeling of cultures differentiated for 3 days in the presence of 10 ng/ml CXCL9, CCL2 or CCL21, compared to basal conditions. Results in A–C are expressed as mean+/−sem from at *n*≥3 independent experiments, *p<0.05, **p<0.01. Scale bar in D, 50 µm.

**Figure 6:**
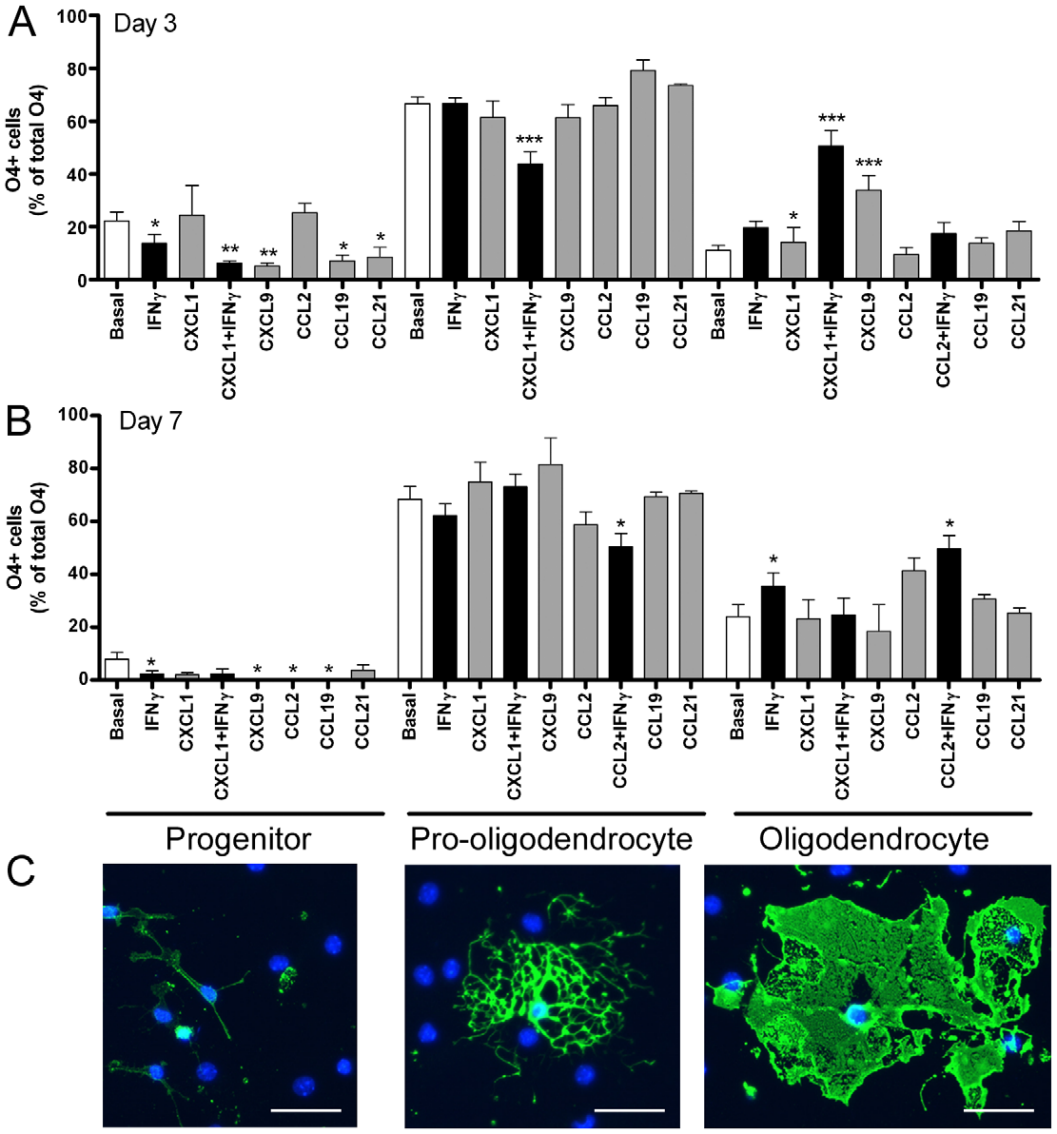
Effect of selected chemokines+/−IFNγ on oligodendrocyte precursor maturation. The effect of chemokines (10 ng/ml)+/−IFNγ (100 U/ml) on oligodendrocyte maturation was assessed by quantifying the percentage of O4 expressing cells at 3 days (A) and 7 days (B) that had the morphology of precursor cells, pro-oligodendrocytes or oligodendrocytes (C). Results are expressed as mean+/−sem from *n* = 3 experiments, *p<0.05, **p<0.01 ***p<0.01. Scale bar in C, 50 µm.

To determine whether CXCL1, CXCL9, CCL2, CCL19 or CCL21 had any effect on NPC differentiation, NPCs from dissociated neurospheres were cultured with chemokines or IFNγ under differentiation conditions and the percentage of neurons, astrocytes and oligodendrocytes was quantitated. Under basal conditions approximately 25% of the cells differentiated into βIII-tubulin positive neurons at 3 days and 65–70% GFAP positive and 8% O4 positive oligodendrocytes at 7 days. While there was some variability in the percentage of differentiated mature cell types in each neurosphere line, in each case CXCL9, CCL2 and CCL21 increased the percentage of neurons compared to basal in each of three independent experiments using different neurosphere lines (ANOVA *F*_(6, 20)_ = 3.035, *p*<0.05) (Fig. 5a) but none of the chemokines tested alone had a significant effect on the percentage of GFAP positive astrocytes (Fig. 5b) or O4 positive oligodendrocytes (Fig. 5c). However, several of the chemokines, particularly when combined with IFNγ, altered the proportion of O4 positive cell subtypes. Based on morphology, the O4 positive cells were classified as precursor cells (bipolar or tripolar cells), pro-oligodendrocytes (a large number of thin processes) or oligodendrocytes (flattened membranous growth) (Fig. 6c). CXCL1 plus IFNγ, as well as CXCL9, reduced the proportion of precursor cells and increased the percentage of oligodendrocytes at day 3 of differentiation (Fig. 6a). By day 7 of differentiation there were very few O4 positive precursor cells remaining and the proportion of oligodendrocytes was increased, which was enhanced by IFNγ alone or by CCL2 plus IFNγ (Fig. 6b).

## Discussion

Chemokines are best known for their chemo-attractant properties and many studies have shown that they promote NPC migration. This has particularly been the case for SDF1/CXL12 and MCP1/CCL2, both *in vitro* [21], [26], [27], [28] and *in vivo* to sites of ischemia [10], [22], [29], [30], traumatic damage [31] or neuroinflammation [7], [32]. Other chemokines have also been shown to regulate NPC migration, including CCL3 and CXCL1 *in vivo* following striatal cell loss [33], CXCL8 and CXCL13 *in vitro* across endothelial cells [34] and CXCL16 regulated migration of a glial precursor cell line [35]. However it is clear that chemokines play a much broader role in the adult nervous system than regulation of NPC migration, under normal physiological conditions and in response to neural disease or injury. We have therefore compared expression of all the CXC and selected CC chemokines, as well as all chemokine receptors, in neurogenic and non-neurogenic regions of the normal adult mouse brain and in adult SVZ-derived neurospheres. As NPCs are involved in CNS injury responses, we have also compared NPC expression levels of chemokines and their receptors in response to the inflammatory cytokines IFNγ and TNFα. We then examined the biological effect on adult neurospheres of CXCL1, CXCL9 and CCL2, whose expression was upregulated by the inflammatory cytokines. To further explore possible non-inflammatory roles of chemokines on NPC biology we examined NPC responses to CXCL5, CXCL8, CXCL16, CCL19, CCL20, CCL21 and CCL25, expression of which was not regulated by inflammatory cytokines.

### Expression of chemokines and receptors in neurogenic and non-neurogenic regions of the CNS

To date, the most comprehensive comparison of chemokine receptor expression in the CNS was performed in embryonic and adult NPCs by Tran and coworkers [8], who showed that these cells expressed most chemokine receptors. They then went on to examine expression of a subset of these receptors in neurogenic regions of the adult mouse, showing that CCR1, CCR2, CCR5, CXCR3 and CXCR4 were expressed [19]. Less information is available on expression of chemokines in the adult CNS, although several are known to be expressed under basal conditions or following neural damage/disease, including MCP1/CCL2, MIP1α/CCL3, GROα/CXCL1, LIX/CXCL5, MCP5/CCL12, and IP10/CXCL10 [36], [37], [38], [39]. Expression of other chemokines, including CCL19, CCL21 and CXCL13, has been described in the CNS, but does not appear to be on CNS parenchymal cells, and rather on infiltrating immune cells or endothelial cells [24].

We have now shown that of all adult neural regions, the olfactory bulb expresses almost all chemokine receptors and most CXC chemokines at the highest levels, with variable expression of receptors in the other neurogenic regions, the SVZ and hippocampus. Non-neurogenic cortex expressed very few of the chemokine receptors and of those that were expressed, they were at low levels compared to the neurogenic brain regions. Unexpectedly, given the relatively restricted expression of the chemokine receptors, there was quite widespread expression of CXC chemokine mRNA, including in cortex. CXCR4 expression could be detected in our cortical samples, indicative of physiological effects of CXCL12, as previously shown [11], [40], [41], [42], [43], [44]. The role of the other CXC chemokines in non-neurogenic cortex under basal conditions is therefore not clear, although they may act on receptors expressed by a small number of cells in the cortex, such as endothelial cells and below the detection limit of RT-PCR under the conditions used. However, this was also observed in SVZ and hippocampus, where chemokines were expressed while their receptors were not, e.g. CXCL1 in hippocampus, which did not express the receptor CXCR2, or CXCL13 in SVZ, which did not express the receptor CXCR5. This may indicate that expression of these chemokines has paracrine effects on surrounding tissue or that there are as yet unidentified ligand/receptor pairings. Conversely, chemokine and cognate receptor expression in the olfactory bulb was widespread and indicates a large physiological role induced by autocrine or paracrine production of chemokines under physiological conditions.

### Regulation of chemokine and receptor expression in adult neurospheres by inflammatory cytokines

IFNγ and/or TNFα did not upregulate expression of any of the chemokine receptors in the neurospheres and, if anything, decreased their expression, particularly for the CC receptors. This is in contrast to astrocytes, where inflammatory stimulation upregulated CCR7 expression [45], but which may have been in response to stimuli other than IFNγ and/or TNFα. Further, other cytokines, such as IL10 and IL-4, upregulate chemokine receptor expression on NPCs [46], indicating that different inflammatory mediators regulate chemokine and chemokine receptor expression in NPCs. Surprisingly, expression of very few of the chemokines examined was upregulated by IFNγ and/or TNFα. The notable exceptions were CXCL1, which was upregulated by TNFα, CXCL9 which was upregulated by IFNγ, CCL2, which was upregulated by IFNγ and TNFα and further enhanced by the combination of the two, and CXCL13, which was only upregulated by a combination of IFNγ and TNFα. Interestingly, CXCL10 and CXCL11 expression was not upregulated in the mouse NPCs, even though, like CXCL9, they are IFNγ inducible in other cell types, such as astrocytes and microglia, which also exhibited cell-type specific differential induction of expression by IFNγ [47]. Therefore, there are cell-type specific responses to inflammatory cytokines in terms of regulation of chemokine expression, indicating possible different functions of these induced chemokines in the different cell types. The inflammatory neural environment could therefore have different effects on NPCs versus their differentiated progeny.

### Regulation of NPC differentiation

Similar to previous reports [22], [23], [48] we found that CCL2 promoted neuronal differentiation. We found that CXCL9 and CCL21 also promoted neuronal differentiation. Given that IFNγ promotes neuronal differentiation of adult NPCs [16] and we show here that IFNγ induces CXCL9, as well as CCL2 expression, it raises the possibility that the effect of IFNγ on neuronal differentiation of NPCs is indirect, through induction of CXCL9 and CCL2. These same chemokines had no effect on astrocyte or total oligodendrocyte numbers. However, they promoted oligodendrocyte maturation, particularly in conjunction with IFNγ. CXCL9 and CXCL1 promoted early oligodendrocyte maturation with effects by day 3 that were gone by day 7, while conversely CCL2 had longer term effects, only promoting oligodendrocyte maturation by day 7. This suggests that their mechanism of action may be different or possibly that early forced maturation of the pro-oligodendrocytes also led to decreased survival of the mature cells. CXCL1 has also been shown to promote oligodendrocyte precursor proliferation [49], [50] and migration arrest [51], with decreased numbers of oligodendrocytes and decreased myelination in CXCR2 null mice [52] and increased numbers and remyelination in mice that overexpress CXCL1 following experimental allergic encephalomyelitis (EAE) [53]. Therefore, CXCL1 plays diverse roles in regulation of oligodendrocyte precursor biology. A role for CXCL9 is less established. CXCR3 null mice show increased demyelination in a model of EAE [54]. This involves regulation of T cells in the nervous system, but in light of the effects of CXCL9 shown here on oligodendrocyte maturation, may also reflect a decreased ability of oligodendrocyte precursor cells to mature as the CXCR3 null cells can no longer respond to the CXCR3 ligand, CXCL9.
